# Effects of rice straw and rice straw ash on rice growth and α-diversity of bacterial community in rare-earth mining soils

**DOI:** 10.1038/s41598-020-67160-w

**Published:** 2020-06-25

**Authors:** Shulan Jin, Wei Jin, Chengxu Dong, Yijun Bai, Decai Jin, Zhongjun Hu, Yizong Huang

**Affiliations:** 1 College of History, Geography and Tourism, Shangrao Normal University, Shangrao, 334000 China; 2 Shangrao Vocational and Technical College, Shangrao, 334109 China; 3 Research Center for Eco-Environmental Sciences, Chinese Academy of Sciences, Beijing, 100085 China; 4 Agro-Environmental Protection Institute, Ministry of Agriculture and Rural Affairs, Tianjin, 300191 China

**Keywords:** Environmental biotechnology, Environmental impact, Soil microbiology

## Abstract

Pot experiments were carried out to study the effects of rice straw (RS) and rice straw ash (RSA) on the growth of early rice and α-diversity of bacterial community in soils around rare earth mining areas of Xunwu and Xinfeng counties in South Jiangxi of China. The results showed that the exploitation of rare earth resources leads to soil pollution around rare earth mining areas and affects the growth of rice, and the content of rare earth elements (REEs) in rice was positively correlated with that in soils and negative correlated with dry weight of rice; The addition of RS to soils around REE mining area can inhibit growth of early rice, and the dry weight of rice grains, shoots, roots is lower when compared with the controls, while the content of REEs is higher. The α-diversity of soil bacterial decreases, which promotes the growth of *Pseudorhodoferax*, *Phenylobacterium* and other bacteria of the same kind, and inhibits the growth of beneficial bacteria. The addition of RSA to soils had no significant effect on α-diversity of soil bacterial but promoted the growth of *Azospira* and other beneficial bacteria, inhibited the growth of *Bryobacter* and other bacteria of the same kind, significantly improved the dry weight of grains, shoots and roots of early rice, and reduced the content of REEs in these parts of rice. It can be concluded that RS is unsuitable to be added to the planting soil of early rice in REE mining area, while RSA is suitable.

## Introduction

Rare earth elements (REEs) widely exist in the lithosphere, biosphere, hydrosphere and even the atmosphere, they migrate and transform in different geochemical environments through ways of precipitation and dissolution, adsorption and desorption, oxidation and reduction, complexation and biological enrichment, thus form different characteristics and patterns of distribution^1–5^. The most important REE minerals are bastnasite, monazite, xenotime, ion-adsorbed rare earth ore and fergusonite. China owns 50% of REE resources and 90% of the ion-type REEs of the whole world, and provides 90% of REE products for the whole world. It is a major country for REE resources, production, export and consumption in the world^6,7^. But the large-scale exploitation and extensive use of REE minerals have led to significant increase of REEs in the environment of mining area and its surroundings, which endangered food security and the health of local residents^8^. Some studies have shown that the accumulation of REEs in the soil is obvious in downwind direction ranges from 8 km to 10 km below the tailings dam of Baotou Iron and Steel Company of China. The content of the mixed REEs in polluted soils near the tailings dam is 27549.58 mg·kg^−1^, 118 times of the CK(control check) and 160 times of the average content of REEs in soils of China^9,10^. It was found that the average content of REEs in the blood and hair of residents in Changting REEs mining area of Fujian province was higher than that of average people, and the exceeded content was 155.6 and 9.6 times of the standard respectively^11,12^. In recent decades, the environmental persistence, bio-accumulation and toxicity of REEs have attracted wide attention^13–15^.

The ionic REEs^16^ are mainly distributed in Southern China (especially Southern Jiangxi province) of the world. Rice is the main crops in this area, and millions of tons of rice straw (RS) is produced when the rice is harvested every year^17^. There are 2 ways for farmers to deal with rice straw, one is to return it to the field directly, the other is to use it as feed or fuel. Crop straw contains certain nutrients and cellulose, hemicellulose, lignin, protein and ash elements. It has not only more organic matter, but also nitrogen, phosphorus, potassium and other nutrients^18,19^. Rice straw incorporation affected physical and chemical properties of soil. Changes in physicochemical properties of soil affect the structure of bacterial community^20,21^. Changes in pH value caused the significant changes of *Acidobacteria* abundance in soil, and the abundance of *Proteobacteria*, *Gemmatimonadetes* and *Nitrospirae* in soil was changed with the changes of nitrogen (N), phosphorus (P) and potassium (K)^22,23^. Soil microorganisms play important parts in decomposing soil organic matter and decontaminating the polluted soil in the whole agricultural ecosystem^18,19,24^. Rice straw incorporation can accelerate the decomposition of organic matter and the transformation of mineral nutrients, increase contents of N, P, K and other elements in soil, and improve the effectiveness of soil nutrients^25^. Rice straw incorporation adds a lot of energy to soil microorganisms, and the number and activity of various microorganisms increase accordingly. Rice straw incorporation can improve microorganisms by 18.9%, contact enzyme activity by 33%, invertase activity by 47%, and urease activity by 17%^19,26^, while some studies also suggest that many organic acids will be produced in the process of decomposition after returning rice straw to the field, which is easy to accumulate in the paddy field. When the concentration becomes high, it will cause harm to the rice growth. Fresh rice straw will produce various organic acids during the process of decomposition, which will be toxic to the roots of crops. When the fresh rice straw is added to the field, microorganisms compete with crops for fertilizer because fresh rice straw contains high content of C and N. Rice straw consumes available nutrients, e.g. Nitrogen in soil during thorough decomposition. The effect of rice straw incorporation on soil microorganisms is closely related to temperature^18^.

The burning of rice straw will produce rice straw ash(RSA). RSA is alkaline and contains potassium oxide, especially silicon dioxide, which reaches more than 70% of the whole substance^27^. Silicon is abundant in soil and considered as a biological stimulant because of its effective function on the growth of different plants^28^. With the intensive development of agriculture, the amount of silicon absorbed from soil increases, which leads to the decrease of available silicon concentration and has potential negative impact on the growth of plants^29^. In 1955, cinders was used as silicate (Si) fertilizer for rice in Korea, Japan and China of East Asia, which leads to a significant increase in rice yield^30^. Rice can accumulate more than 10% silicon in buds, which is necessary to maintain the healthy growth and stable high yield of rice. The application of silicon-rich materials will affect the dynamic changes of different elements in soils^31^. The effective function of Si include: improving the oxidizing capacity of roots, thereby reducing the absorption of Fe and Mn, preventing fungal and insect attacks; improving the effectiveness of phosphorus (P)^32^. The addition of Si in the polluted soil could effectively reduce the disease index of tomatoes, affected the abundance of microorganisms and enzyme activity in soils^29,33,34^. The microbial biomass and available nutrient content of rice soils were both improved after 2 years of continuous application of Si fertilizer^35^. Microorganism converts polymeric Si into monomeric Si, which can provide stable mineral surface or gel structure for organisms, e.g. diatoms, plants, bacteria and fungi. On the other hand, single cell microorganism plays a key role in the biological absorption and transportation of Si^35^.

Rice straw incorporation can form dissolved organic matter (DOM) in rice soil^36–38^. DOM can affect the bio-availability of heavy metals in two distinct ways. DOM is a negatively charged colloid, which competes with heavy metals in the soil for adsorption sites. It can promote the dissolution and desorption of REEs and other heavy metals in the soil and become the “carrier” for the migration and activation of heavy metals such as REEs. DOM contains a large number of carboxyl, hydroxyl, carbonyl and other functional groups, which have the ability to become complexation with heavy metals. Organic matter may also directly or indirectly interact with heavy metals through “bridges” of Fe or S, thus reduce the mobility of heavy metals in soil^39^. Many studies have shown that silicate ions can react with REEs to form precipitation of rare earth silicate compounds when silicon-containing materials were added to soil. The application of silicon-containing materials in soil can increase the pH value of soil, enhance the adsorption capacity of soil, adsorb rare earth ions with opposite charges in soil, and reduce the activity of REEs^40–43^. Silicon is an important element of cell wall. Silicon binds with cadmium (Cd) and REEs, and adheres to the cell wall. The addition of Silicon can enhance the interception of Cd and REEs by cell wall^44^.

Microorganisms play an important part in regulating energy and nutrient flux in soil, thus affects soil function and productivity^45^. Because adding RS and RSA to soils changed soil nutrients, affected the bio-availability of REEs in soil, and also affected rice growth and structure microbial communities. This effect is affected by many factors, e.g. season, temperature, humidity and so on^46^. For example, in the long-term production practice, farmers have found that rice straw incorporation can indeed improve rice yield, but there are seasonal differences in the effect of rice straw incorporation on rice yield. When rice straw is applied to the early rice, rice tends to grow poorly, however, when RS is applied to the late rice, the rice tends to grow better, and the reasons are still unclear till now.

Therefore, the purpose of this research is to study the effect of different type of rice straw incorporation on the interaction among plants, microorganisms and soil, reveal the microbiological mechanism of rice straw and rice straw ash returning to field on improving soil productivity of paddy field, thus provide theoretical and practical guidance for the safe production of rice in rare earth mining areas, the rational utilization of renewable resources in rural areas and the protection of ecological environment.

## Results

### Effects of RS and RSA on the biomass and REE contents in different part of rice

**Figure 1:**
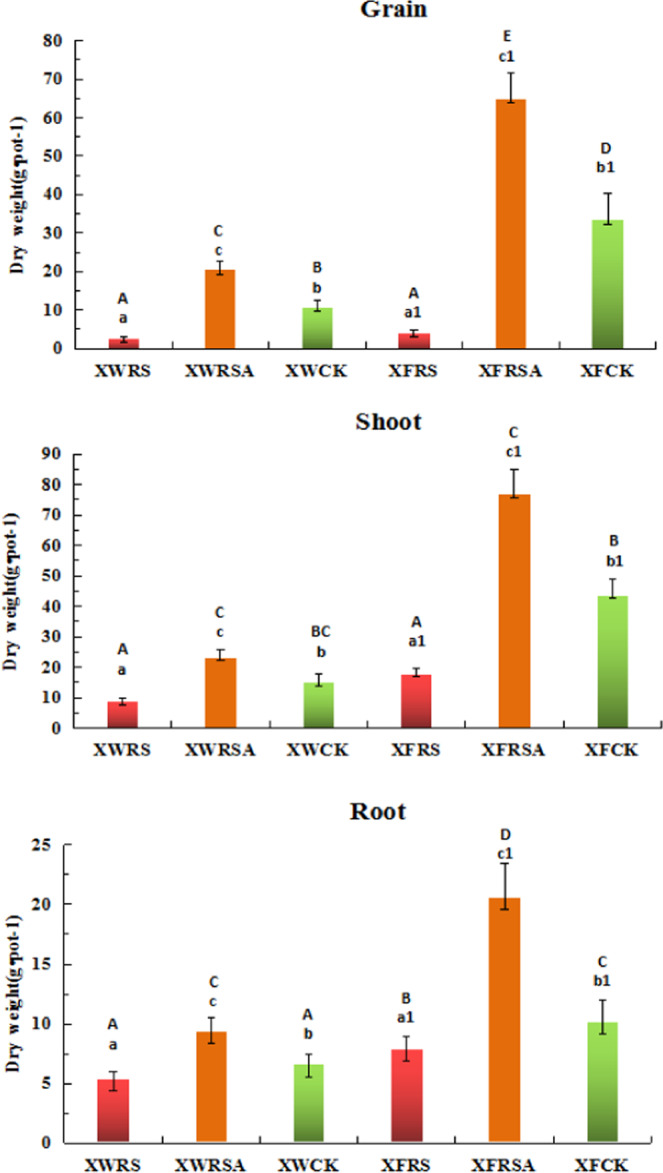
Effects of RS and RSA on biomass of rice grains, roots and shoots. Note: Each data measured three times and the average value was taken, a, b, c. refers to the significant difference of dry weight of rice in Xunwu mining area, a1, b1, c1. refers to the significant difference of dry weight of rice in Xinfeng mining area, and A,B,C,D. refers to the significant difference of dry weight of rice in all experimental plots of both two mining areas. (n = 3,p < 0.05).

It can be seen from Fig. 1 that the addition of RS to the polluted soil inhibited the rice growth in mining areas of both Xunwu and Xinfeng counties, and the dry weight of grains, shoots and roots of early rice was lower when compared with CK; the addition of RSA to the polluted soil could effectively improve the growth of early rice, and dry weight of grain, shoots and roots of early rice was apparently higher when compared with CK. The dry weight of rice grain, shoot and root decreased by 74.8%, 41.8% and 17.7% respectively with the addition of RS to the polluted soils around Xunwu mining area, while increased by 90.8%, 54.4% and 41.9% respectively with the addition of RSA to the polluted soils. In Xinfeng mining area, the dry weight of grains, shoots and roots of early rice in the polluted soils added with RS was 87.7%, 59.1% and 22.5% lower when compared with CK, while the dry weight of rice in polluted soils added with RSA was 94%, 76.2% and 103% higher respectively when compared with CK.

**Figure 2:**
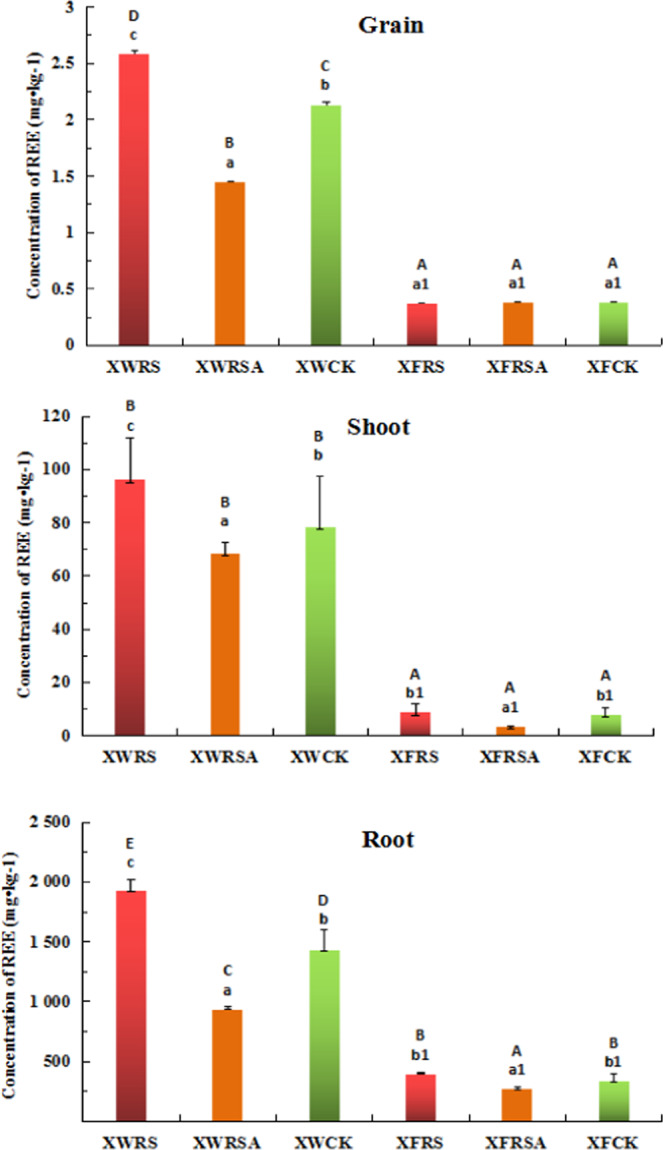
Effects of RS and RSA on total REE concentration in rice grains, roots and shoots. Note: Each data measured three times and the average value was taken, a, b, c. refers to the significant difference of total REE concentration of rice in Xunwu mining area, a1, b1. refers to the significant difference of total REE concentration of rice in Xinfeng mining area, and A,B,C,D. refers to the significant difference of dry weight of rice in all experimental plots of both two mining areas. (n = 3,p < 0.05).

As can be seen from Fig. 2 that RS promoted rice to uptake and accumulate more REEs. The concentrations of REEs in grains, shoots and roots of rice added RS in soils of Xunwu mining area are 78.3%, 22.5% and 34.8% higher respectively when compared with CK. The addition of RSA inhibited the absorption and accumulation of REEs in rice. The concentrations of REEs in grains, shoots and roots of early rice are 32.0%, 12.6%, 34.3% lower respectively when compared with CK; The concentration of REEs in shoots and roots of rice added RS in soils of Xinfeng mining area was increased by 8.7% and 17.9% respectively when compared with CK, but the effect of RS on rice grain was not obvious. The concentrations of REEs in rice shoots and roots was inhibited with the addition of RSA, which was 56.8% and 19.7% lower respectively when compared with CK.

As can be seen from Table S2 that the pH value in soil of Xunwu mining area added with RS was 6.70 as that of CK, and the soil pH of Xunwu mining area added with RSA was increased by 4.63% when compared with CK. The soil pH of Xinfeng mining area added with RS or RSA was increased by 1.39% and 1.62% respectively when rice matured.

### Effects of RS and RSA on α-diversity of soil bacterial community

**Figure 3:**
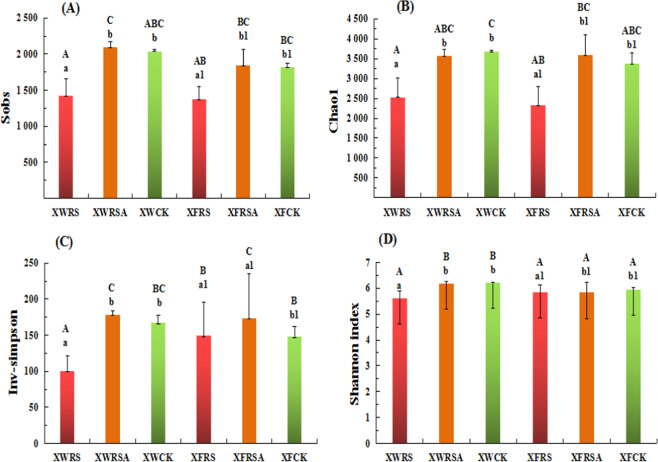
Effects of RS and RSA on α-diversity of soil bacterial community. Note: Each data measured three times and the average value was taken, a, b. refers to the significant difference of α-diversity of soil bacterial community in Xunwu mining area, a1, b1. refers to the significant difference of α-diversity of soil bacterial community in Xinfeng mining area, and A,B,C. refers to the significant difference of α-diversity of soil bacterial community in all experimental plots of both two mining areas. (n = 3,p < 0.05).

The rarefaction curves slopes were near the saturation, which meant the sequencing depth was reasonable (Figure S1). It was observed that the indexes of sobs, chao, inv-simpson and Shannon of bacterial community decreased by 30.4%, 31.0%, 39.8% and 9.6% respectively when rice straw was added to soils around Xunwu REE mining area. The sobs and chao of bacterial community decreased by 24.5% and 31.3% respectively when RS was added to the polluted soils around Xinfeng REE mining area, which had no significant effect on indexes of inv-simpson and Shannon. Whether in soils of Xunwu or Xinfeng REE mining area, the addition of RSA had no significant effect on bacterial communities such as indexes of sobs, chao, inv-simpson and Shannon (Fig. 3).

### Effects of RS and RSA on structure of soil bacterial community

**Figure 4:**
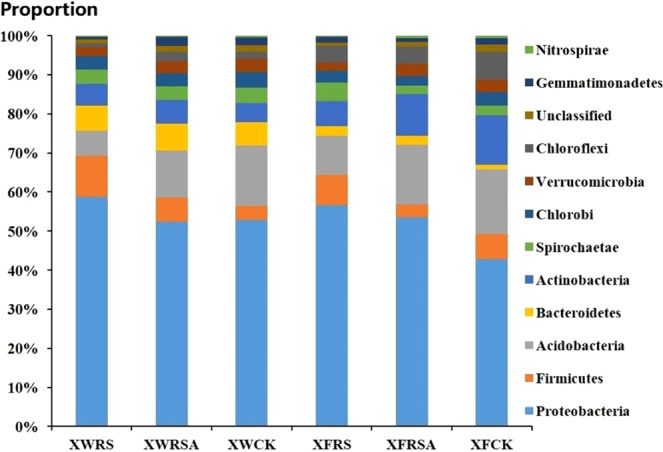
Effects of rice straw and rice straw ash on the level of Phylum of bacterial community.

Under the condition of 97% similarity degree, the comparison between OTU and NCBI Greengene database showed that the samples could be annotated at 5 taxonomic levels: phylum, class, order, family and genus. The results showed that the relative abundance of *Proteobacteria* and *Firmicutes* with the addition of RS and RSA in soils of Xunwu REE mining area was higher than that of CK, *Acidobacteria*, *Chloroflexi*, *Gemmatimonadetes*, *Verrucomicrobia* and *Nitrospirae* were lower than that of CK, and the relative abundance of *Firmicutes*, *Bacteroides*, *Actinobacteria*, *Chloroflexi*, *Gemmatimonadetes* with the addition of RSA were higher than that of CK, *Acidobacteria* and *Verrucomicrobia* were lower than that of CK. The relative abundance of *Proteobacteria*, *Firmicutes*, *Bacteroidetes* and *Spirochaetae* with the addition of RS in soils of Xinfeng REE mining area was higher than that of CK, while the relative abundance of *Acidobacteria*, *Chlorobi*, *Actinobacteria*, *Verrucomicrobia*, *Gemmatimonadetes* and *Nitrospirae* was lower than that of CK; the relative abundance of *Proteobacteria*, *Bacteroidetes* with the addition of RSA was lower than that of CK. The relative abundance of *Firmicutes*, *Actinobacteria*, *Chlorobi*, *Chloroflexi* and *Gemmatimonadetes* was lower than that of CK.(Fig. 4).

**Figure 5:**
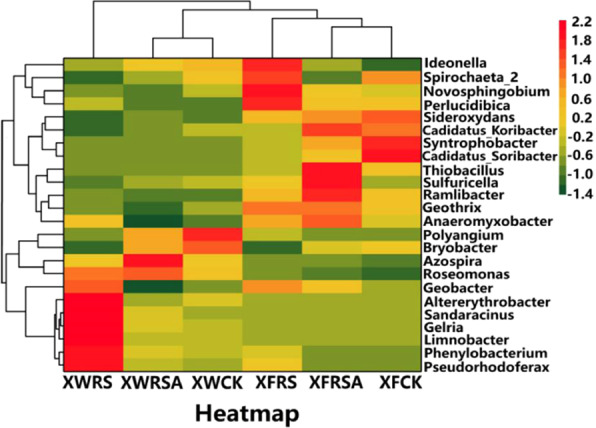
The Heat-map of the top 24 genera in polluted soils around REE mining area.

As can be seen from Fig. 5 that the dominant bacteria in soils of Xunwu REEs mining area with the addition of RS are *Pseudorhodoferax*, *Phenylobacterium*, *Limnobacter*, *Altererythrobacter*, *Gelria*, *Sandaracinus*, *Geobacter*, *Roseomonas*, *Perlucidibaca*, accounting for 4.4%, 3.8%, 2.2%, 1.6%, 1.6%, 1.6%, 2.2%, 2.3% and 1.1%, of the whole bacterial community respectively. The relative abundance is higher when compared with CK, and the relative abundance of *Bryobacter*, *Candidus_Koribatacter*, *Spribibaca_2* was significantly lower when compared with CK; *Azospira* and *Roseomonas* that added RSA in soils were the dominant bacteria, accounting for 1.6% and 2.5% of the whole bacterial community respectively. Relative abundance of *Azospira* and *Roseomonas* was higher when compared with CK, while *Bryobacter* abundance was lower when compared with CK, and the relative abundance of most bacteria changed little, similar to that of CK. *Perlucidibaca*, *Novosphingobium*, *Geothrix*, *Sulfuricella*, *Geobacter* and *Ideonella* were dominant bacteria in soils of Xinfeng REE mining area with the addition of RS, accounting for 3.8%, 3.2%, 2.5%, 2.3%, 2.1% and 2.1% of the whole bacterial community respectively. The relative abundance of *Pseudorhoferax*, *Phenylobacterium* was also apparently higher when compared with CK. The relative abundance of *Candidus_Korib*, *Syntrophoter*, *Bobacter* were also significantly lower when compared with CK. the relative abundance of *Sulfuricella*, *Ramlibacter* and *Thiobacillus* added RSA was higher when compared with CK, but the relative abundance of *Spirochaeta_2* was lower than that of CK. The relative abundance of most bacteria changed little, which was similar to that of CK.

### Direct and indirect effects of environmental factors on soil bacterial communities

Table 1 shows that sobs, chao, inv-simpson and Shannon of bacterial communities in soils of Xunwu REE mining area by three treatments have strong positive correlation with the dry weight of rice grains (GW), shoots (SW) and roots (RW), negatively correlated with the content of REEs in rice grains (GR), shoots (SR), roots (RR), and positively correlated with the soil pH at maturity stage(pHM). GW, SW and RW were negatively correlated with GR, SR and RR, and positively correlated with pHM. As can be seen from Table 2 that sobs and chao of the soil bacterial communities in Xinfeng rare earth mining area by three treatments were positively correlated with GW and SW, positively correlated with RW; inv-simpson was weakly positively correlated with GW, and extremely correlated with SW and RW; Shannon was weakly positively correlated with GW, but had little correlation with SW and RW; GR was positively correlated with sobs, chao and Shannon, but no correlation with inv-simpson; SR was slightly correlated with sobs and chao, but negatively correlated with inv-simpson. RR was negatively correlated with sobs, chao and inv-simpson, but no correlation with Shannon; pHM was less correlated with sobs and chao, positively correlated with inv-simpson and negatively correlated with Shannon; GR was positively correlated with GW, SW and RW, SR was negatively correlated with GW, SW and RW, RR was negatively correlated with GW, SW and RW; pHM was not significantly correlated with GW, SW, RW, but negatively correlated with GR and SR, and had little correlation with RR.

**Table 1:** Correlation among rice dry weight, rare earth, water-soluble REE Concentration, soil pH and bacterial α-diversity of Xunwu mining area.

	sobs	chao	invsimpson	shannon	GW	SW	RW	GR	SR	RR	pHM
sobs	1	0.988	0.998^*^	0.993	0.872	0.862	0.773	−0.84	−0.957	−0.899	0.557
chao		1	0.976	0.999^*^	0.786	0.773	0.666	−0.746	−0.9	−0.821	0.422
invsimpson			1	0.983	0.902	0.893	0.813	−0.873	−0.973	−0.926	0.61
shannon				1	0.806	0.794	0.69	−0.768	−0.914	−0.84	0.452
GW					1	0.988	0.985	−0.998^*^	−0.977	−0.998^*^	0.892
SW						1	0.988	−0.999^*^	−0.972	−0.997	0.901
RW							1	−0.994	−0.924	−0.973	0.957
GR								1	0.962	0.993	−0.918
SR									1	0.988	−0.775
RR										1	−0.864
pHM											1

* At 0.05 level (double tail), the correlation was significant, n = 3.

**Table 2:** Correlation among rice dry Weight, rare earth, water-soluble REE Concentration, soil pH and bacterial α-diversity of Xinfeng mining area.

	Sobs	Chao	Invsimpson	Shannon	GW	SW	RW	GR	SR	RR	pHM
sobs	1	0.993	0.498	0.413	1.0^*^	0.851	0.671	0.87	−0.274	−0.873	−0.346
chao		1	0.596	0.303	0.99	0.906	0.753	0.806	−0.385	−0.924	−0.234
invsimpson			1	−0.584	0.478	0.88	0.977	0.005	−0.971	−0.858	0.641
shannon				1	0.433	−0.128	−0.399	0.809	0.763	0.083	−0.997^*^
GW					1	0.839	0.654	0.881	−0.253	−0.862	−0.367
SW						1	0.96	0.48	−0.739	−0.999^*^	0.199
RW							1	0.217	−0.897	−0.947	0.464
GR								1	0.236	−0.519	−0.764
SR									1	0.708	−0.807
RR										1	−0.155
pHM											1

*At 0.05 level (double tail), the correlation was significant, n = 3.

## Discussion

The exploitation of rare earth minerals has affected soil bacterial community. *Polyangium* and *Bryobacter* are dominant bacteria in XWCK soil, which account for 2.5% and 2.3% of the whole bacterial community respectively. *Polyangium* is a type of bacteria producing highly active anticancer substances^47^. *Bryobacter* is positively correlated with the incidence of crop Fusarium wilt^48^. *Candidatus_Solibacter* and *Syntrophobacter* are dominant bacteria in XFCK soil, which account for 4.7% and 3.1% of the whole bacterial community respectively. *Candidatus_Solibacter* is related to the degradation of woodland into grassland caused by exploitation of rare earth resources^49^. *Syntrophobacter* is a hydrogen-producing and acetic acid-producing functional bacteria, which may be related to the sewage environment produced by exploitation of rare earth resources^50^. When the farmland is polluted by rare earth, which not only affects the soil bacterial community, but also affects the growth of rice. The rare earth reserves, development scale and coverage area of tailings in Shipai mining area of Xunwu are significantly larger than those in Longshe mining area of Xinfeng, and the environmental pollution is more serious than that in Longshe mining area. The concentrations of REEs in rice grains, shoots and roots in Shipai mining area are significantly higher than those in Longshe mining area, while the dry weight of each part is significantly lower than that in Longshe mining area (Fig. 3), which indicates that the content of REEs in rice is positively correlated with the concentration of REEs in soil and negatively correlated with the dry weight of rice. It was reported that with the exploitation of rare earth minerals in southern Jiangxi of China, the acidity of soil gradually increases, its specific adsorption of REEs was weakened. REEs have the tendency of conversion from residue form to active form, which leads to the decrease of the ratio of residual REEs. The ratio of non-residue REEs in soil to total REEs is over 80%. The dissolved REEs are easily absorbed and transported to the above ground by plants^26^. This study indicated that the content of REEs in rice grains, shoots and roots of Shipai mining area was very high without the addition of RS and RSA in the polluted soils especially when the REE content in rice grains exceed national safety standard for rice of 2.0 mg/kg in China, which threatened people’s health.

Rice straw incorporation can improve the content of carbon (C) and nitrogen (N) in soil, thus affecting community structure of soil bacterial. The abundance of *Proteobacteria* increased after the rice straw was added to the soils of the two mining areas due to the increase of N content in soil^23^. *Nitrospirae* likes to grow under the condition of low nitrogen (N) and low carbon (C). The increase of N and C content in soils causes the decline of abundance of *Nitrospirae*. The addition of RS affected physical and chemical properties of soils and changed community structure of bacterial^51^. The relative abundance of *Seudorhodoferax*, *Phenylobacterium*, *Limnobacter*, *Altererythrobacter*, *Gelria*, *Sandaracinus*, *Geobacter*, *Roseomonas* and *Perlucidibaca* in soils of Xunwu mining area with the addition of RS was significantly higher than that of CK. Studies indicated that *Pseudorhodoferax* was related to the activation of lactose phosphate; *Phenylobacterium* may be a harmful bacterium which can affects the growth of crops; The relative abundance of *Limnobacte* and *Gelria* was related to organic pollutants; *Sandaracinus* was related to plant residual soil^52–54^. The abundance of *Perlucidibaca*, *Novosphingobium*, *Geothrix*, *Sulfuricella*, *Geobacter* and *Ideonella* in Xinfeng REE mining area with the addition of RS in soils were significantly higher than that of CK. *Novosphingobium* was positively correlated with CO_2_ concentration, and the change of relative abundance of *Ideonella* and *Geobacter* was correlated with the change of soil physical and chemical properties and content of organic matter^55,56^. The *Bryobacter* of the two mining areas was lower than that of CK with the addition of RS in soils, which may be related to FA enrichment^48^. The growth period of rice in this experiment is mainly in June and July, when the temperature becomes higher and the light become stronger, the rice straw decomposes rapidly. Soil eutrophication promotes the growth of harmful bacteria and inhibits the growth of beneficial bacteria e.g. *Nitrospirae*^18,51^, *Nitrospirae* affects urease, ammonia monooxygenase and hydroxylamine dehydrogenase, and improves the efficiency of nitrogen fertilizers e.g. urea^57^. The decrease of its abundance is one of the reasons for the significant reduction of rice yield in two mining areas, thus the content of REEs in rice roots, shoots and grains increased due to the significant decrease in dry weight of rice.

The addition of RSA can improve Si content in soil, thus affects the community structure of bacteria. The *Azospira* and *Roseomonas* in the soil of Xunwu mining area added RSA was significantly higher than that of CK. The range of growth temperature and PH conditions of *Azospira* were much wider. Under the suitable conditions, *Azospira* had higher nitrogenase activity and phosphorus solubility, thus it had the function of promoting crop growth. The increase of *Roseomonas* abundance was due to the improvement of soil nutrients^58^. The increased abundance of these classes may be beneficial for the soil quality. *Bryobacter* was positively correlated with the incidence of crop diseases, and its reduction could reduce the incidence of rice. The relative abundance of *Sulfuricella*, *Ramlibacter* and *Thiobacillus* added RSA in soils of Xinfeng mining area was higher than that of CK. *Thiobacillus* has biological phosphorus removal capability^59^. Its increase may be due to the abundant available P in RSA. *Sulfuricella* was also affected by the distribution of available P^60^. *Spirochaeta_2* exists in the activated sludge and sewage, and its reduction may be due to the optimization of soil environment with the addition of RSA, which caused its abundance to be lower than the control. Therefore, the addition of RSA can increase the abundance of beneficial bacteria and reduce the abundance of harmful bacteria, which is one of the reasons for the improvement of rice yield. In addition, Si itself is beneficial to plant growth and is considered as a bio-stimulant, which is the second reason for the improvement of rice yield. Si reduces the bio-availability of REEs in soil, which may be the third reason for the improvement of rice yield. The REE content in rice decreases due to the decrease of bio-availability of REEs in soil and the increase of dry weight of rice.

## Conclusion

**Figure 6:**
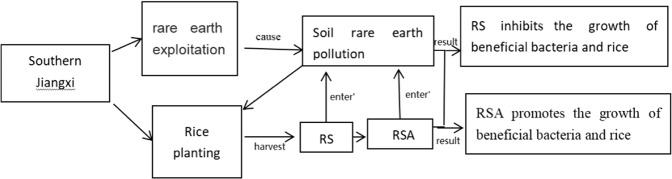
Flow chart of the influence of RS and RSA on rice planting in the rare earth polluted area of South Jiangxi Province in China.

The excessive exploitation of rare earth can lead to soil pollution in mining area and its surroundings, thus affect normal growth of early rice. The content of REEs in rice is positively correlated with the content of REEs in soil and negatively correlated with the dry weight of rice. Adding RS to the polluted soil promotes the dissolution of REEs and inhibits the growth of early rice due to the decrease of pH value in the first stage, while in the second stage, high temperature and strong light makes RS decompose rapidly, which causes soil eutrophication to be more easy. The indices of sobs, Chao, inv-simpson and Shannon of bacterial community all decreased, and soil eutrophication promoted the growth of harmful bacteria, it also inhibited the growth of beneficial bacteria and early rice, thus the dry weight of grains, shoots and roots of rice is apparently lower, while the REEs content is apparently higher when compared with CK. RSA is alkaline, so the addition of RSA can improve pH value of soil, reduce the activity of REEs in soil, reduce the absorption of rare earth by rice. It has no significant effect on α-diversity of bacterial community, but can promote the growth of beneficial bacteria, and significantly improve rice yield (Fig. 6).

## Materials and methods

### Soil collection and preparation of related materials

**Figure 7:**
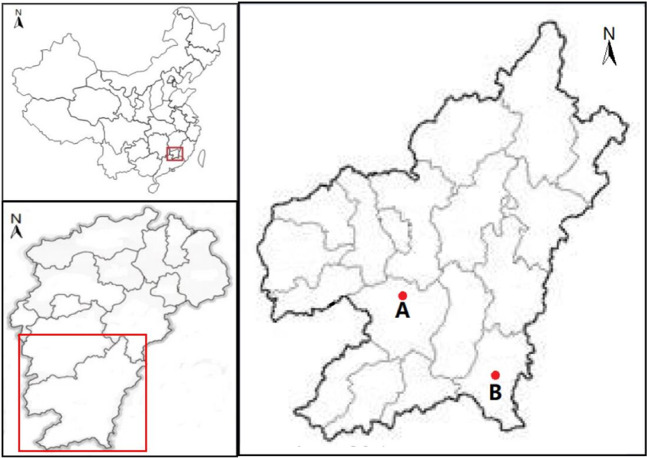
Distribution map of Sampling Points in Southern Jiangxi of China. Notes: “A” stands for Longshe Village, Jiading Town of Xinfeng County; “B” stands for Shipai Village, Wenfeng Town of Xunwu County. (The map is drawn by arcGIS 10.7, and the website of GIS software is: https://www.esrichina.com.cn/ArcGIS/arcgis10_7.html).

Soil cultivation of rice was conducted in typical mining areas of Shipai Village, Wenfeng Township of Xunwu County, which is typical light REE mining area, and Longshe Village, Jiading Town of Xinfeng County, which is typical heavy REE mining area (Fig. 7). The surface soil of 20 cm was collected and air-dried through 100-mesh sieve. The soil was stored at room temperature of dry environment. The tested RS was collected from Yujiang District of Yingtan City, Jiangxi Province, where there was no REEs pollution. Meanwhile, the RS was used to prepare RSA for reserve, and the RS was crushed into powder by a grinder. The physicochemical properties of soil, RS and RSA and their REE concentrations are shown in Table S1.

Pot experiments were carried out to study the effects of different amendments on the ability of REE absorption and accumulation in different part of early rice. Three treatments were set: adding 2.0% RS, adding 2.0% RSA and CK (untreated soil), each treatment was repeated for three times. In the experiment, a plastic pot with diameter of 26 cm and height of 28 cm was used and each pot was filled with 5 kg of soil, 5.4 g of urea (containing 46% N) and 0.6 g of Potassium chloride (containing 63% K_2_O) were used as fertilizers for the growth of rice (Jin *et al*., 2019). According to the design, different amendments were mixed with soil and evenly packed into a plastic basin. The balance of flooding is maintained for 2 weeks, and the water level maintained at about 3 cm.

The rice variety used in the experiment was Zhongjiazao 17, which was sourced from Jiang**xi** Keyuan Seed Industry Co., Ltd. The average growing period of this type of rice is 109 days, which has wide adaptability and a large planting area in Jiangxi Province. The seeds were disinfected with 10% H_2_O_2_ solution for 10 minutes, then rinsed by water for many times. After germination, the seeds were sown to the soil without REEs pollution for seedling raising. After 3 weeks of growth, the seedlings of early rice were transplanted into the cultivation pots, and each pots for two seedlings. The pot experiment was carried out in the conditions of greenhouse. The daily light intensity, duration of light, temperature and humidity of the greenhouse were adjusted to those of Ganzhou REEs mining area of Jiangxi Province till the rice ripen.

After maturation, rice roots, shoots and millet were harvested, washed by deionized water, and placed in the indoor ventilation area for natural air-drying till the weight keeps constant, hull was removed and the unpolished rice was collected, then the unpolished rice, roots and shoots were powdered and stored in the condition of room temperature. When rice was harvested, soils in the pots was collected, some of which was used for measuring pH value, the others was placed in a refrigerator at −80 °C, which was used for molecular experiments.

### Chemical analysis

Soil pH was determined by composite electrode potentiometry, and cation exchange capacity (CEC) by sodium acetate-flame photometry, the total P by NaOH melting-molybdenum antimony colorimetry, total C, total N and total S by elemental analyzer (Elementar, Vario Macro, Germany). The particle size of soil was detected by Laser Particle Analyzer of Mastersizer 2000 Type (Malvern Instruments, UK).

The 0.25 g soil sample was weighed in a 100-mL digestion tube (three replicates for each sample), 5 mL aqua regia (HNO_3_:HCl = 3:1) was added, balanced overnight at room temperature, then heated to 120 °C for 12 hours, and then heated to 140 °C until the color of soil turned white. The digested sample was cooled and volatilized acid in fume hood, then volumed to 50 ml by the pure water and filtered by filter membrane of 0.45 um, then the total contents of 15 rare earth elements (Y, La, Ce, Pr, Nd, Sm, Eu, Gd, Tb, Dy, Ho, Er, Tm, Yb, Lu) were determined by inductively coupled plasma mass spectrometry (ICP-MS, 7500, Agilent Technologies). Quality control was maintained with GBW07405 from National Research Center for Certified Reference Materials (Beijing, China).

0.2 g powder of unpolished rice, 0.2 g power of rice shoot and 0.1 g power of rice root were placed in 50-ml polyethylene centrifugal tube respectively (three replicates per sample), soaked with 3 ml of high-quality pure nitric acid for whole night, all samples were digested by microwave (MARS, Matthew Inc., USA), and the digestion procedure was referred to Jin *et al*., 2019. The whole process of sample digestion was controlled by GBW08502 of China’s National Standard Substance, then the digested samples were put in airing chamber for cooling and acid removal, then the volume was set to 50 ml and passed through a 0.45 µm filter, and the total amount of 15 rare earth elements (ibid.) was determined by inductively coupled plasma mass spectrometry (ICP-MS).

### Amplification and gene sequencing for 16S r RNA

In this experiment, Fast DNA Spin Kit (MP Biomedical Company, US) was used to extract total DNA from soil microorganisms. After the extraction, the total DNA of soil microorganisms was detected by 1% agarose electrophoresis, and the concentration was determined by nucleic acid analyzer of NanoDrop 2000 (Thermo Scientific, USA). Quantitative PCR of 16S rRNA gene of bacteria in this experiment was performed with universal bacterial primers. The specific sequence of primers was 338 F: ACTCCTACGGGAGGCAGCAG(5′−3′) and 518 R: ATTACCGCGGCTGC TGG (5′−3′). The Q-PCR in this experiment was carried out on the platform of LightCycler 480(Roche Applied Science). The reaction procedure was: 95 °C for 30 s, 95 °C for 5 s, 60 °C for 30 s, 50 °C for 30 s, and the cycle repeated 30 times. After the amplification, the number of copies of the sample was calculated according to the standard curve drawn from the standard sample data. 16S rRNA V4 region of bacteria was amplified by bacterial specific primers 515 F (5′-GTGCCAGCMGCCGCGGGGTAA-3′) and 806 R (5′-GGACTACHVGGTWTCTAAT-3′). The primer sequence of each sample contained 6 bp of specific tag sequence (Barcode) to distinguish different samples. Reaction procedure: 95 °C for 5 minutes, 95 °C for 30 seconds, 50 °C for 30 seconds, 72 °C for 30 seconds, 72 °C for 10 minutes. The amplified products of PCR were identified and separated by 1% agarose gel electrophoresis. The PCR products were purified by DNA Agarose Purification Kit (Agarose Gel DNA Purification Kit, TaKaRa). The purified products were submitted to Beijing Xinke open source bioinformatics Illumina MiSeq sequencing platform for further sequencing.

The Illumina MiSeq sequencing results were analyzed by QIIME software, eliminating sequences shorter than 200 bp, and then eliminating chimera sequence information by Uchime and algorithm software to obtain high-quality sequences. The valid sequences are clustered into OTU (Operational Taxonomic Unit) according to 97% consistency. The original sequence obtained by sequencing was submitted to NCBI database and the sequence number was obtained: SRP190113.

### Statistical analysis

Bacteria richness indices of Sobs, Chao1 and diversity indices of Shannon and invsimpson were calculated by QIIME. Among them, Sobs and Chao1 were used to estimate the relative abundance of samples. The larger the value is, the greater the relative abundance of the community becomes. The indices of Shannon and inv-simpson were used to estimate the microbial diversity in the samples. The larger the value is, the higher the community diversity becomes. Excel 2010 and SPSS 17.0 software were used for statistical analysis of all basic data. Analysis of significance was performed by the method of ANOVA, and Pearson correlation was used to determine the correlation tests between α-diversities and environment factors; software of Origin 9.0 was used to draw the pictures.

## Supplementary information

Supplementary Information.
