# Supplementary material for: Effects of rice straw and rice straw ash on rice growth and α-diversity of bacterial community in rare-earth mining soils
Source: Sci Rep. 2020 Jun 25;10:10331. doi: 10.1038/s41598-020-67160-w (PMC7316728; doi:10.1038/s41598-020-67160-w)
Supplement: Supplementary file 1 — Supplementary Information. [file 41598_2020_67160_MOESM1_ESM.doc]

**Effects of rice straw and rice straw ash on rice growth and α-diversity of bacterial community in rare-earth mining soils**

Shulan Jin^1^,Wei Jin^2^, Chengxu Dong^1^, Yijun Bai^1^, Decai Jin^3^, Zhongjun Hu^1^*, Yizong Huang^4^*

**Table S1, S2 and Figure S1**

**Table S1 Physicochemical properties and REE concentrations of soil, RS and RSA**

| Items | XW Soil | XF Soil | RS | RSA |
| --- | --- | --- | --- | --- |
| pH | 5.54 | 5.55 | 5.85 | 11.93 |
| CEC (cmol/kg) | 5.85 | 7.75 | - | - |
| Organic matter (g/kg) | 1.89 | 2.17 | - | - |
| Total N (g/kg) | 1.11 | 1.10 | - | - |
| Total C (g/kg) | 12.51 | 10.98 | - | - |
| Total S (g/kg) | 0.35 | 0.49 | - | - |
| Total P(g/kg) | 0.25 | 0.31 | 0.68 | 5.13 |
| Soil particle size- Clay (%) | 20.62 | 15.76 | - | - |
| Silt (%)  Sand (%) | 67.42  11.96 | 69.40  14.86 | -  - | -  - |
| Y (mg/kg) | 34.31 | 62.43 | 0.26 | 2.4 |
| La (mg/kg) | 252.56 | 161.13 | 0.63 | 5.16 |
| Ce (mg/kg) | 293.13 | 176.49 | 0.52 | 4.28 |
| Pr (mg/kg) | 72.39 | 42.39 | 0.19 | 1.32 |
| Nd (mg/kg) | 151.91 | 102.42 | 0.44 | 3.88 |
| Sm (mg/kg) | 28.23 | 20.51 | 0.12 | 1.04 |
| Eu (mg/kg) | 4.08 | 4.51 | 0.08 | 0.36 |
| Gd (mg/kg) | 9.18 | 11.45 | 0.12 | 0.88 |
| Tb (mg/kg) | 2.76 | 3.31 | 0.06 | 0.24 |
| Dy (mg/kg) | 3.31 | 7.79 | 0.07 | 0.6 |
| Ho (mg/kg) | 2.57 | 3.58 | 0.03 | 0.2 |
| Er (mg/kg) | 6.08 | 9.98 | 0.04 | 0.44 |
| Tm (mg/kg) | 0.80 | 1.4 | 0.03 | 0.2 |
| Yb (mg/kg) | 4.88 | 8.86 | 0.05 | 0.36 |
| Lu (mg/kg) | 0.7 | 1.25 | 0.03 | 0.12 |
| Total REEs (mg/kg) | 866.93 | 617.47 | 2.67 | 21.48 |

**Table S2 The effect of rice straw and rice straw ash on pH of soil**

| XWRS | XWRSA | XWCK | XFRS | XFRSA | XFCK |
| --- | --- | --- | --- | --- | --- |
| 6.70 | 7.01 | 6.70 | 6.06 | 6.08 | 5.98 |

**
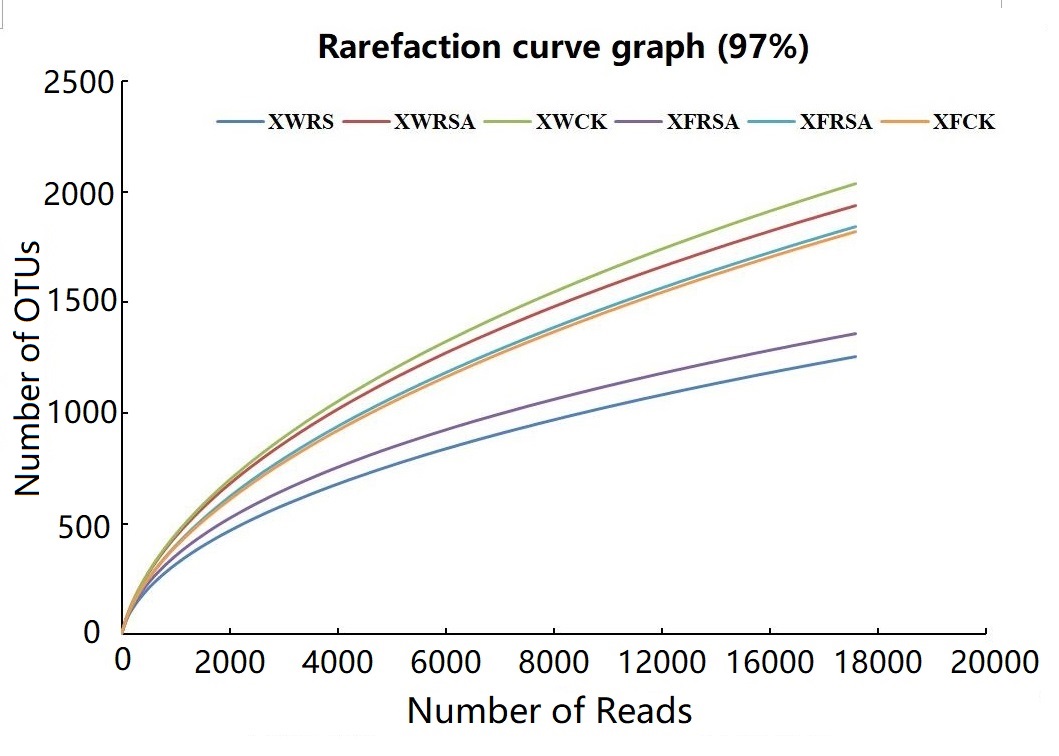
**

**Figure S1 Rarefaction curves of bacterial 16S gene sequences.**

(OTU is the abbreviation of operational taxonomic unit)
